# Liberal Versus Restrictive Blood Transfusion Strategies in Neurocritical Care: A Systematic Review and Meta‐Analysis of Randomized Controlled Trials

**DOI:** 10.1155/ccrp/6179847

**Published:** 2026-01-12

**Authors:** Ayesha Shaukat, Muhammad Ahmed Zahoor, Komal Khan, Aiman Shahid Khan, Rubaisha Saleem, Anupama Ariyasiri, Syed Abdul Aziz Jameel, Shahab Afridi, Syeda Javeria Salman, Noor Naeem, Marib Ashraf, Amamah Rauf Chaudhry, Zobia Ahmad, Muhammad Omar Larik, Muhammad Hasanain, Muhammad Umair Anjum, Aymar Akilimali

**Affiliations:** ^1^ Department of Medicine, Dow Medical College, Karachi, Pakistan, duhs.edu.pk; ^2^ Department of Medicine, Ziauddin University, Karachi, Pakistan, zu.edu.pk; ^3^ Department of Medicine, Dow International Medical College, Karachi, Pakistan, duhs.edu.pk; ^4^ Department of Medicine, Ayub Medical College, Abbottabad, Pakistan, ayubmed.edu.pk; ^5^ Department of Medicine, Rashid Latif Medical College, Lahore, Pakistan, rlmclahore.com; ^6^ Department of Medicine, Mayo Clinic, Phoenix, Arizona, USA, mayo.edu; ^7^ Department of Research, Medical Research Circle, Goma, Democratic Republic of the Congo

**Keywords:** anemia, blood transfusion, liberal strategy, neurocritical care, restrictive strategy

## Abstract

**Introduction:**

Neurocritical care patients, including those with traumatic brain injury, subarachnoid hemorrhage, and intracerebral hemorrhage, often develop anemia, compromising brain oxygen delivery and increasing morbidity and mortality. Blood transfusion strategies, either liberal or restrictive, are commonly used to manage anemia in these patients, but the optimal approach remains unclear due to mixed results in existing studies.

**Methods:**

A systematic search of PubMed, Cochrane Library, ScienceDirect, and Google Scholar from inception to December 2024 for randomized controlled trials (RCTs) evaluating restrictive versus liberal transfusion strategies in adult neurocritical care patients. Outcomes included mortality, Glasgow Outcome Scale (GOS), red blood cell (RBC) units transfused, sepsis, intensive care unit (ICU)/hospital length of stay, and secondary complications. The study is registered with PROSPERO (CRD42025635426).

**Findings:**

The analysis included seven RCTs with 1941 patients. The restrictive strategy significantly reduced the number of RBC units transfused per patient (MD: 2.36; 95% CI: 1.08–3.64; *p* = 0.0003) and was associated with a lower incidence of sepsis (RR: 0.73; 95% CI: 0.56–0.96; *p* = 0.02). There were no significant differences between restrictive and liberal strategies for ICU (RR 0.74; 95% CI 0.28–1.91; *p* = 0.53), in‐hospital (RR 0.77; 95% CI 0.35–1.68), 30‐day (RR 0.91; 95% CI 0.70–1.18), 6‐month (RR 0.98; 95% CI 0.67–1.44), or long‐term mortality (RR 1.00; 95% CI 0.80–1.24). GOS scores at 6 months showed no significant difference (RR 0.94; 95% CI 0.83–1.07). ICU and hospital length of stay were also comparable between strategies. Secondary outcomes, including stroke, brain hypoxia, intracranial hypertension, and other non‐neurological complications, showed no significant differences between the two strategies.

**Conclusion:**

Restrictive transfusion strategies are as effective as liberal strategies in terms of mortality and neurological complications, with additional benefits such as fewer RBC transfusions and lower sepsis rates. These findings support restrictive strategies as a safer approach to managing anemia in neurocritical care, though further research on long‐term outcomes is needed.

**Highlights**

◦ Neurocritical care patients are at high risk of anemia, which can compromise brain oxygen delivery and worsen outcomes. Blood transfusion strategies vary, with restrictive and liberal approaches debated for their safety and efficacy.
◦ This meta‐analysis includes seven RCTs with 1941 patients, demonstrating that a restrictive transfusion strategy reduces transfusion volume and the risk of sepsis.
◦ Mortality, ICU length of stay, and neurological outcomes showed no significant differences between the two strategies.
◦ A restrictive transfusion approach may offer a safer alternative without compromising patient outcomes.
◦ Further large‐scale studies are needed to refine transfusion thresholds and identify patient subgroups that may benefit most.

## 1. Introduction

Neurocritical care patients, such as those suffering from traumatic brain injury (TBI), subarachnoid hemorrhage (SAH), and intracerebral hemorrhage (ICH), commonly develop anemia. This condition compromises oxygen delivery to the brain, significantly increasing both morbidity and mortality in these critically ill patients [1–3]. Studies report that anemia occurs in 40–60% of patients with SAH, with the CONSCIOUS‐1 trial highlighting that a hemoglobin (Hb) level < 10 g/dL was independently associated with poor neurological outcomes and increased mortality [4]. In another large cohort of 640 patients, nearly half of SAH patients became anemic during their ICU stay, and those with more severe anemia had a significantly higher risk of delayed cerebral ischemia and poor functional recovery at 6 months [5]. Furthermore, 45.5% of patients with TBI and 25.8% of individuals with ICH during their hospital stay also develop anemia [6–8].

The brain typically compensates for anemia by increasing cardiac output, redirecting blood flow to prioritize cerebral perfusion, inducing vasodilation to enhance cerebral blood flow, activating physiological mechanisms to effectively reduce blood viscosity, and increasing cerebral oxygen extraction. However, these compensatory mechanisms can be impaired in the setting of brain injury due to disrupted cerebral autoregulation, hemodynamic instability, or acute heart failure. As a result, patients remain at risk for anemia‐induced secondary brain injury, even at relatively higher Hb concentrations [9–11].

Clinicians must choose between liberal and restrictive blood transfusion strategies to manage anemia among neurocritical patients. A liberal transfusion strategy aims to maintain higher Hb levels, typically by initiating transfusion when Hb falls below 10.0 g/dL and maintaining levels between 10.0 and 12.0 g/dL [12, 13]. This approach ensures adequate tissue oxygenation, potentially improves cognitive function, and reduces the risk of secondary brain injury. However, liberal transfusion may also increase the risk of transfusion‐related complications, including elevated intracranial pressure, acute lung injury, acute respiratory distress syndrome (ARDS), and circulatory overload [12–14].

Conversely, a restrictive transfusion strategy involves initiating transfusion only when Hb falls below 7.0 g/dL, with target maintenance levels between 7.0 and 9.0 g/dL [12, 15]. This approach reduces transfusion frequency and minimizes transfusion‐associated risks. However, a compromised oxygen delivery potentially impairs cognitive function and increases the risk of secondary brain injury [16–18]. Currently, the optimal transfusion strategy for neurocritical care patients remains unclear. Existing studies yield mixed results, with some trials assessing unfavorable neurological outcomes at 6 months and reporting no significant difference between liberal and restrictive transfusion strategies [19]. Moreover, current data on long‐term functional and patient‐centered outcomes related to transfusion strategies in critically ill adults are insufficient to inform clinical practice [3, 20]. Given the complexity of these cases, a more individualized approach that considers patient‐specific factors such as age, illness severity, comorbidities, and the balance of potential risks and benefits is likely necessary.

This meta‐analysis seeks to systematically compare the outcomes of liberal and restrictive transfusion strategies to determine which approach is better suited for improving clinical outcomes in neurocritical patients. A deeper understanding of the underlying mechanisms and a comprehensive analysis of available research are essential to refine anemia management guidelines in neurocritical care.

## 2. Methodology

This systematic review and meta‐analysis followed the “Preferred Reporting Items for Systematic Review and Meta‐Analyses” (PRISMA) guidelines [21]. The protocol of this study was registered in the International Prospective Register of Systematic Reviews (PROSPERO) with the reference number CRD42025635426.

### 2.1. Data Sources and Search Strategy

A comprehensive and systematic search was conducted to identify studies meeting the established eligibility criteria from inception (i.e., the start of indexing for each database) till December 2024. For additional information, the reference lists of all eligible studies were meticulously reviewed. The search terms included relevant PubMed MeSH terms and related keywords, such as Traumatic Brain Injury (TBI), Subarachnoid Hemorrhage (SAH), Anemia, Blood Transfusion, Restrictive Transfusion Strategy, and Liberal Transfusion Strategy. Search strategies utilized for each database are outlined in Supplementary Table 1.

### 2.2. Study Selection and Eligibility Criteria

All articles retrieved from the systematic search were imported into the EndNote reference library, Version X8.1 (Clarivate Analytics), where duplicates were removed. Two authors (A.S. and M.A.Z.) independently reviewed and selected studies, with any disagreements resolved by a third author (A.S.K.). Studies that met the preliminary criteria were retrieved for a full‐text review to confirm their relevance based on the inclusion criteria.

Predetermined criteria were established to ensure the inclusion of relevant studies in this systematic review and meta‐analysis. The inclusion criteria for the studies were as follows: (i) studies reporting neurocritical patients, including those with TBI, SAH, or ICH, who experienced anemia during their intensive care unit (ICU) stay; (ii) studies presenting comparisons between liberal red blood cell (RBC) transfusion strategy and restrictive RBC transfusion strategy; (iii) studies reporting at least one of the outcomes of interest; and (iv) published randomized controlled trials (RCTs). Only RCTs were included to minimize bias and ensure direct comparisons between transfusion strategies. The RBC transfusion threshold was defined according to the criteria set by the authors of each included study. The exclusion criteria were as follows: (i) studies published in languages other than English and (ii) case reports, case series, editorials, commentaries, letters, meta‐analyses, conference abstracts, opinion pieces, and other non‐peer‐reviewed publications.

### 2.3. Data Extraction

Two authors (M.A.Z. and R.S.) independently evaluated the data and supplementary materials, with any discrepancies resolved through consultation with a third author (A.S.). The following data were extracted from the included studies: (i) baseline clinical characteristics of the study populations; (ii) primary outcomes, including mortality rates (ICU, hospital, 30‐day, 6‐month, and long‐term), unfavorable Glasgow Outcome Scale (GOS) scores, the number of RBC units transfused per patient, blood transfusion data during ICU stay (patients transfused and patients developing infections), and length of stay (ICU and hospital); and (iii) secondary outcomes, which included adverse neurological and non‐neurological events.

In line with our PROSPERO registration, the primary outcomes were mortality and unfavorable neurological outcomes (defined as GOS ≤ 3). Additional transfusion‐related, clinical, and complication outcomes were analyzed as secondary or exploratory endpoints and should be interpreted as such.

### 2.4. Quality Assessment and Risk of Bias

Risk of bias and quality assessment of the RCTs was executed by two independent reviewers (S.A.A.J. and S.A.) utilizing the Cochrane Risk of Bias Tool for Randomized Controlled Trials (RoB‐2) [22]. All studies underwent a comprehensive screening process and were subsequently classified as exhibiting either a “low risk,” “moderate risk,” or “high risk” of bias. Any discrepancies were addressed and resolved via mutual discussion by a third reviewer (A.S.).

Furthermore, to ensure a rigorous methodological approach in this systematic review and meta‐analysis, the quality of evidence was assessed using the GRADE (Grading of Recommendations Assessment, Development, and Evaluation) framework through the GRADEpro GDT software (Supplementary Table 2) [23].

### 2.5. Statistical Analysis

Two authors (A.S.K. and R.S.) conducted the statistical analysis using Review Manager software (RevMan Version 5.4; Copenhagen: The Nordic Cochrane Center, The Cochrane Collaboration, 2020). The Mantel–Haenszel random‐effects model was applied to aggregate dichotomous outcomes, calculating risk ratios (RRs). The inverse‐variance random‐effects model was used to calculate standardized differences (SDs) for continuous outcomes. The 95% confidence intervals (CI) were calculated for all outcomes, and a *p*‐value of ≤ 0.05 was deemed statistically significant. Statistical heterogeneity was evaluated using the I^2^ index, with values < 50%, 50%–75%, and > 75% indicating low, moderate, and high heterogeneity, respectively. To identify studies contributing to significant heterogeneity, a sensitivity analysis was conducted using a leave‐one‐out approach. Additionally, subgroup analyses were conducted to explore the effects of transfusion strategies on specific outcomes.

## 3. Results

### 3.1. Literature Search and Characteristics of Studies

**Figure 1:**
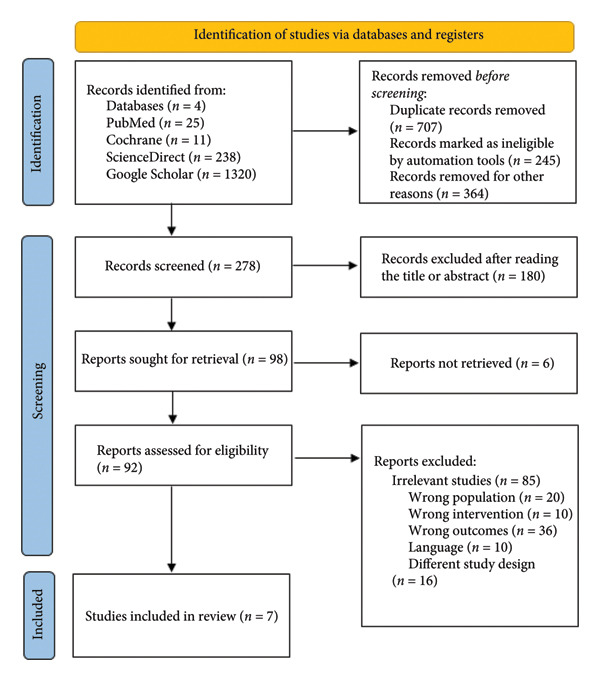
PRISMA flow diagram of the number of studies screened and included in the systematic review.

An extensive systematic search was conducted across four databases (PubMed, Cochrane Library, ScienceDirect, and Google Scholar), initially identifying 1594 potentially relevant studies. After excluding duplicates and conducting thorough title, abstract, and full‐text screening, seven studies were deemed eligible for inclusion in this systematic review and meta‐analysis [12, 13, 15, 19, 24–26]. The detailed process of selection and exclusion is depicted clearly in the PRISMA flow diagram (Figure 1).

The selected studies were all RCTs. A total of 1941 patients were included, with 977 participants assigned to the liberal strategy group and 964 to the restrictive strategy group. A total of five studies included patients with TBI [12, 13, 19, 24, 26], one study involved patients with a combination of TBI, SAH, or ICH [15], and one study focused solely on patients with SAH [25]. The ages of participants varied across the studies, with a mean age of 44.5 ± 15.6 years for the liberal strategy group and 44.6 ± 13.7 years for the restrictive strategy group. The proportion of male and female participants was also noted, with 1274 males and 664 females across the included studies. A detailed overview of the baseline demographics and outcomes is presented in Tables 1 and 2.

**Table 1:** Baseline characteristics of the included studies.

Author, year	Mortality rate *n*/*N* (%)										Length of stay (days, mean ± SD)				Unfavorable GOS outcomes—at 6 months *n*/*N* (%)		No. RBC transfusions (units per patient, mean ± SD)		Blood transfusions during ICU admission *n*/*N* (%)				Neurological adverse events *n*/*N* (%)												Non‐neurological outcomes *n*/*N* (%)																	
	30 days		60 days		6 months		ICU		Hospital		ICU (days)		Hospital (days)						Patients transfused		Patients developing infection		Vasospasm		Stroke		Intracranial hypertension requiring therapy		Brain tissue hypoxia		Ventriculitis, meningitis, or brain abscess		Seizures		Thromboembolic events		Hypotension		ARDS		Sepsis		UTI		Bacteremia		Pneumonia		Pulmonary embolus		Acute myocardial infarction	
	Liberal strategy	Restrictive strategy	Liberal strategy	Restrictive strategy	Liberal strategy	Restrictive strategy	Liberal strategy	Restrictive strategy	Liberal strategy	Restrictive strategy	Liberal strategy	Restrictive strategy	Liberal strategy	Restrictive strategy	Liberal strategy	Restrictive strategy	Liberal strategy	Restrictive strategy	Liberal strategy	Restrictive strategy	Liberal strategy	Restrictive strategy	Liberal strategy	Restrictive strategy	Liberal strategy	Restrictive strategy	Liberal strategy	Restrictive strategy	Liberal strategy	Restrictive strategy	Liberal strategy	Restrictive strategy	Liberal strategy	Restrictive strategy	Liberal strategy	Restrictive strategy	Liberal strategy	Restrictive strategy	Liberal strategy	Restrictive strategy	Liberal strategy	Restrictive strategy	Liberal strategy	Restrictive strategy	Liberal strategy	Restrictive strategy	Liberal strategy	Restrictive strategy	Liberal strategy	Restrictive strategy	Liberal strategy	Restrictive strategy
Turgeon et al. 2024	—	—	—	—	99/369 (26.8%)	96/365 (26.3%)	63/369 (17.1%)	56/367 (15.3%)	85/369 (23.0%)	79/367 (21.5%)	15 ± 8.89	15 ± 8.89	33 ± 23.70	33 ± 26.67	170/239 (71.1%)	182/242 (75.2%)	4.11 ± 2.22	0.84 ± 0.74	365/369 (98.9%)	141/367 (38.4%)	204/369 (55.3%)	192/367 (52.3%)	—	—	—	—	—	—	—	—	12/369 (3.3%)	15/367 (4.1%)	—	—	—	—	—	—	—	—	21/369 (5.7%)	28/367 (7.6%)	—	—	24/369 (6.5%)	27/367 (7.4%)	28/369 (7.6%)	121/367 (33.0%)	—	—	—	—
Taccone et al. 2024	82/397 (20.6%)	94/418 (22.5%)	—	—	—	—	—	—	—	—	21.4 ± 15.7	22.5 ± 15.6	42.0 ± 34.8	45.5 ± 39.3	246/393 (62.6%)	300/413 (72.6%)	2.29 ± 1.48	0.88 ± 0.74	357/397 (89.9%)	205/423 (48.5%)	—	—	—	—	35/397 (8.8%)	57/423 (13.5%)	—	—	5/397 (1.3%)	3/423 (0.7%)	—	—	—	—	19/397 (4.8%)	17/423 (4.0%)	42/397 (10.6%)	40/423 (9.5%)	29/397 (7.3%)	36/423 (8.5%)	45/397 (11.3%)	64/423 (15.1%)	—	—	—	—	—	—	—	—	0/397 (0%)	1/423 (0.2%)
Gobatto et al. 2019	—	—	—	—	2/21 (9.5%)	7/23 (30.4%)	1/21 (4.8%)	7/23 (30.4%)	1/21 (4.8%)	7/23 (30.4%)	21 ± 15.56	16 ± 3.70	35 ± 31.11	42 ± 39.26	8/21 (38.1%)	13/23 (56.5%)	3.1 ± 1.6	1.5 ± 1.7	21 (100%)	13 (57%)	—	—	4/21 (19.0%)	15/23 (65.2%)	2/21 (9.5%)	0/23 (0%)	20/21 (95.2%)	23/23 (100%)	—	—	1/21 (4.8%)	1/23 (4.3%)	0/21 (0%)	1/23 (4.3%)	3/21 (14.3%)	0/23 (0%)	18/21 (85.7%)	20/23 (86.9%)	1/21 (4.8%)	1/23 (4.3%)	7/21 (33.3%)	14/23 (60.9%)	3/21 (14.3%)	0/23 (0%)	—	—	5/21 (23.8%)	9/23 (39.1%)	1/21 (4.8%)	1/23 (4.3%)	0/21 (0%)	0/23 (0%)
Robertson et al. 2014	—	—	—	—	17/101 (16.8%)	14/99 (14.1%)	—	—	—	—	—	—	—	—	63/94 (67.0%)	50/87 (57.5%)	—	—	73/101 (72.3%)	52/99 (52.5%)	36/101 (35.6%)	27/99 (27.3%)	—	—	2/101 (2.0%)	1/99 (1.0%)	43/101 (42.6%)	39/99 (39.4%)	26/101 (25.7%)	31/99 (31.3%)	3/101 (3.0%)	3/99 (3.0%)	7/101 (6.9%)	4/99 (4.0%)	22/101 (21.8%)	8/99 (8.1%)	28/101 (27.7%)	32/99 (32.3%)	25/101 (24.8%)	16/99 (16.2%)	5/101 (5.0%)	3/99 (3.0%)	6/101 (6.0%)	7/99 (7.1%)	2/101 (2.0%)	2/99 (2.0%)	20/101 (19.8%)	13/99 (13.1%)	6/101 (6.0%)	1/99 (1.0%)	1/101 (1.0%)	1/99 (1.0%)
Naidech et al. 2010	—	—	—	—	—	—	—	—	—	—	—	—	—	—	—	—	—	—	20/21 (95.2%)	19/23 (82.6%)	—	—	5/21 (23.8%)	5/23 (21.7)	6/21 (28.6%)	9/23 (39.1%)	—	—	—	—	—	—	—	—	—	—	1/21 (4.8%)	1/23 (4.3%)	3/21 (14.3%)	8/23 (34.8%)	—	—	—	—	—	—	2/21 (9.5%)	2/23 (8.7%)	—	—	—	—
Zygun et al. 2009	—	—	—	—	—	—	10%	—	15%	—	23	—	—	—	4 (21%)	—	—	—	—	—	—	—	—	—	—	—	—	—	—	—	—	—	—	—	—	—	—	—	—	—	—	—	—	—	—	—	—	—	—	—	—	—
McIntyre et al. 2006	5/38 (13.1%)	5/29 (17.2%)	5/38 (13.2%)	5/29 (17.2%)	—	—	3/38 (7.9%)	3/29 (10.3%)	5/38 (13.2%)	5/29 (17.2%)	8 ± 4.44	10 ± 11.85	30.5 ± 22.22	27 ± 18.52	—	—	4.6 ± 2.5	1.4 ± 2.2	100%	59%	2/38 (5%)	2/29 (7%)	—	—	—	—	—	—	—	—	—	—	—	—	—	—	—	—	—	—	—	—	—	—	—	—	—	—	—	—	—	—

*Note:* Data are expressed as *n*/*N* (%) unless otherwise specified. Mortality, unfavorable GOS outcomes, transfusion rates, infections, neurological events, and non‐neurological complications are reported as the number of patients with the event over total patients (*n*/*N*), with percentages in parentheses. Length of stay is reported in days as mean ± standard deviation (SD). The number of red blood cell (RBC) transfusions is reported as mean ± SD units per patient. HTN = hypertension.

Abbreviations: ARDS = acute respiratory distress syndrome; GOS = Glasgow Outcome Scale; ICU = intensive care unit; MI = myocardial infarction.

**Table 2:** Primary and secondary outcomes reported in the included studies.

Author, year	Mortality rate *n*/*N* (%)										Length of stay (days, mean ± SD)				Unfavorable GOS outcomes—at 6 months *n*/*N* (%)		No. RBC transfusions (units per patient, mean ± SD)		Blood transfusions during ICU admission *n*/*N* (%)				Neurological adverse events *n*/*N* (%)												Non‐neurological outcomes *n*/*N* (%)																	
	30 days		60 days		6 months		ICU		Hospital		ICU (days)		Hospital (days)						Patients transfused		Patients developing infection		Vasospasm		Stroke		Intracranial hypertension requiring therapy		Brain tissue hypoxia		Ventriculitis, meningitis, or brain abscess		Seizures		Thromboembolic events		Hypotension		ARDS		Sepsis		UTI		Bacteremia		Pneumonia		Pulmonary embolus		Acute myocardial infarction	
	Liberal strategy	Restrictive strategy	Liberal strategy	Restrictive strategy	Liberal strategy	Restrictive strategy	Liberal strategy	Restrictive strategy	Liberal strategy	Restrictive strategy	Liberal strategy	Restrictive strategy	Liberal strategy	Restrictive strategy	Liberal strategy	Restrictive strategy	Liberal strategy	Restrictive strategy	Liberal strategy	Restrictive strategy	Liberal strategy	Restrictive strategy	Liberal strategy	Restrictive strategy	Liberal strategy	Restrictive strategy	Liberal strategy	Restrictive strategy	Liberal strategy	Restrictive strategy	Liberal strategy	Restrictive strategy	Liberal strategy	Restrictive strategy	Liberal strategy	Restrictive strategy	Liberal strategy	Restrictive strategy	Liberal strategy	Restrictive strategy	Liberal strategy	Restrictive strategy	Liberal strategy	Restrictive strategy	Liberal strategy	Restrictive strategy	Liberal strategy	Restrictive strategy	Liberal strategy	Restrictive strategy	Liberal strategy	Restrictive strategy
Turgeon et al. 2024	—	—	—	—	99/369 (26.8%)	96/365 (26.3%)	63/369 (17.1%)	56/367 (15.3%)	85/369 (23.0%)	79/367 (21.5%)	15 ± 8.89	15 ± 8.89	33 ± 23.70	33 ± 26.67	170/239 (71.1%)	182/242 (75.2%)	4.11 ± 2.22	0.84 ± 0.74	365/369 (98.9%)	141/367 (38.4%)	204/369 (55.3%)	192/367 (52.3%)	—	—	—	—	—	—	—	—	12/369 (3.3%)	15/367 (4.1%)	—	—	—	—	—	—	—	—	21/369 (5.7%)	28/367 (7.6%)	—	—	24/369 (6.5%)	27/367 (7.4%)	28/369 (7.6%)	121/367 (33.0%)	—	—	—	—
Taccone et al. 2024	82/397 (20.6%)	94/418 (22.5%)	—	—	—	—	—	—	—	—	21.4 ± 15.7	22.5 ± 15.6	42.0 ± 34.8	45.5 ± 39.3	246/393 (62.6%)	300/413 (72.6%)	2.29 ± 1.48	0.88 ± 0.74	357/397 (89.9%)	205/423 (48.5%)	—	—	—	—	35/397 (8.8%)	57/423 (13.5%)	—	—	5/397 (1.3%)	3/423 (0.7%)	—	—	—	—	19/397 (4.8%)	17/423 (4.0%)	42/397 (10.6%)	40/423 (9.5%)	29/397 (7.3%)	36/423 (8.5%)	45/397 (11.3%)	64/423 (15.1%)	—	—	—	—	—	—	—	—	0/397 (0%)	1/423 (0.2%)
Gobatto et al. 2019	—	—	—	—	2/21 (9.5%)	7/23 (30.4%)	1/21 (4.8%)	7/23 (30.4%)	1/21 (4.8%)	7/23 (30.4%)	21 ± 15.56	16 ± 3.70	35 ± 31.11	42 ± 39.26	8/21 (38.1%)	13/23 (56.5%)	3.1 ± 1.6	1.5 ± 1.7	21 (100%)	13 (57%)	—	—	4/21 (19.0%)	15/23 (65.2%)	2/21 (9.5%)	0/23 (0%)	20/21 (95.2%)	23/23 (100%)	—	—	1/21 (4.8%)	1/23 (4.3%)	0/21 (0%)	1/23 (4.3%)	3/21 (14.3%)	0/23 (0%)	18/21 (85.7%)	20/23 (86.9%)	1/21 (4.8%)	1/23 (4.3%)	7/21 (33.3%)	14/23 (60.9%)	3/21 (14.3%)	0/23 (0%)	—	—	5/21 (23.8%)	9/23 (39.1%)	1/21 (4.8%)	1/23 (4.3%)	0/21 (0%)	0/23 (0%)
Robertson et al. 2014	—	—	—	—	17/101 (16.8%)	14/99 (14.1%)	—	—	—	—	—	—	—	—	63/94 (67.0%)	50/87 (57.5%)	—	—	73/101 (72.3%)	52/99 (52.5%)	36/101 (35.6%)	27/99 (27.3%)	—	—	2/101 (2.0%)	1/99 (1.0%)	43/101 (42.6%)	39/99 (39.4%)	26/101 (25.7%)	31/99 (31.3%)	3/101 (3.0%)	3/99 (3.0%)	7/101 (6.9%)	4/99 (4.0%)	22/101 (21.8%)	8/99 (8.1%)	28/101 (27.7%)	32/99 (32.3%)	25/101 (24.8%)	16/99 (16.2%)	5/101 (5.0%)	3/99 (3.0%)	6/101 (6.0%)	7/99 (7.1%)	2/101 (2.0%)	2/99 (2.0%)	20/101 (19.8%)	13/99 (13.1%)	6/101 (6.0%)	1/99 (1.0%)	1/101 (1.0%)	1/99 (1.0%)
Naidech et al. 2010	—	—	—	—	—	—	—	—	—	—	—	—	—	—	—	—	—	—	20/21 (95.2%)	19/23 (82.6%)	—	—	5/21 (23.8%)	5/23 (21.7)	6/21 (28.6%)	9/23 (39.1%)	—	—	—	—	—	—	—	—	—	—	1/21 (4.8%)	1/23 (4.3%)	3/21 (14.3%)	8/23 (34.8%)	—	—	—	—	—	—	2/21 (9.5%)	2/23 (8.7%)	—	—	—	—
Zygun et al. 2009	—	—	—	—	—	—	10%	—	15%	—	23	—	—	—	4 (21%)	—	—	—	—	—	—	—	—	—	—	—	—	—	—	—	—	—	—	—	—	—	—	—	—	—	—	—	—	—	—	—	—	—	—	—	—	—
McIntyre et al. 2006	5/38 (13.1%)	5/29 (17.2%)	5/38 (13.2%)	5/29 (17.2%)	—	—	3/38 (7.9%)	3/29 (10.3%)	5/38 (13.2%)	5/29 (17.2%)	8 ± 4.44	10 ± 11.85	30.5 ± 22.22	27 ± 18.52	—	—	4.6 ± 2.5	1.4 ± 2.2	100%	59%	2/38 (5%)	2/29 (7%)	—	—	—	—	—	—	—	—	—	—	—	—	—	—	—	—	—	—	—	—	—	—	—	—	—	—	—	—	—	—

*Note:* Data are expressed as *n*/*N* (%) unless otherwise specified. Mortality, unfavorable GOS outcomes, transfusion rates, infections, neurological events, and non‐neurological complications are reported as the number of patients with the event over total patients (*n*/*N*), with percentages in parentheses. Length of stay is reported in days as mean ± standard deviation (SD). The number of red blood cell (RBC) transfusions is reported as mean ± SD units per patient. HTN = hypertension.

Abbreviations: ARDS = acute respiratory distress syndrome; GOS = Glasgow Outcome Scale; ICU = intensive care unit; MI = myocardial infarction.

### 3.2. Quality Assessment of Included Studies

A comprehensive evaluation using RoB‐2 was conducted across all included studies, encompassing both those from the previous meta‐analysis and those published subsequently. The assessment revealed that two studies met all criteria, showing no concerns in any domain [19, 26]. Two studies exhibited some concerns [13, 15], and three studies were classified as having a high risk of bias [12, 24, 25]. Specifically, one study showed deviations from the intended interventions (D2), and three others raised concerns in this area. The same study also demonstrated risks in the selection of the reported result (D5), a concern shared by two other studies. Furthermore, only one study exhibited bias in the measurement of the outcome (D4). A graphical summary of the risk of bias is provided in Supplementary Figure 1.

#### 3.2.1. Primary Outcomes

##### 3.2.1.1. Mortality Rates (ICU, Hospital, 30‐Day, 6‐Month, and Long‐Term)

**Figure 2:**
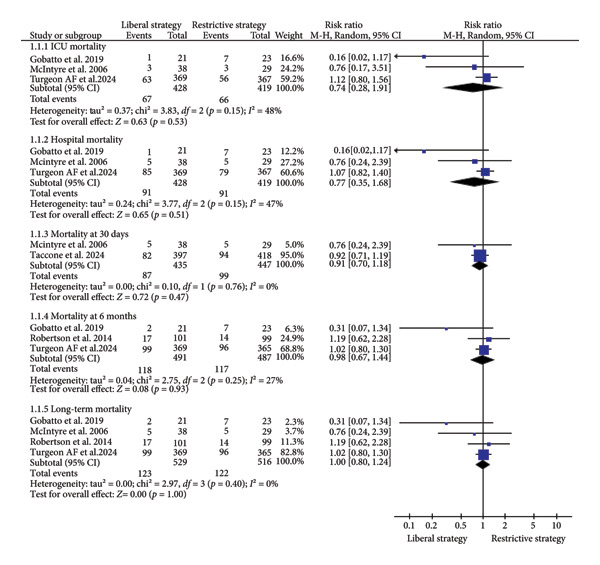
The impact of different transfusion strategies on mortality rates at ICU, hospital, 30‐day, 6‐month, and long‐term.

A total of three studies (*n* = 847) reported on ICU mortality, with results indicating no significant difference between the restrictive and liberal strategy groups (RR = 0.74; 95% CI: 0.28 to 1.91; *p* = 0.53; I^2^ = 48%; Figure 2) [12, 19, 24]. Similarly, three studies (*n* = 847) evaluating in‐hospital mortality also showed no significant difference between the two groups (RR = 0.77; 95% CI: 0.35 to 1.68; *p* = 0.51; I^2^ = 47%; Figure 2) [12, 19, 24]. Regarding 30‐day mortality, 2 studies (*n* = 882) found no significant difference between the restrictive and liberal strategies (RR = 0.91; 95% CI: 0.70 to 1.18; *p* = 0.47; I^2^ = 0%; Figure 2) [12, 15]. For the 6‐month mortality, three studies (*n* = 978) similarly reported no significant difference between the two groups (RR = 0.98; 95% CI: 0.67–1.44; *p* = 0.93; I^2^ = 27%; Figure 2) [13, 19, 24]. Finally, four studies (*n* = 1045) assessing long‐term mortality also revealed no significant difference between the restrictive and liberal strategy groups (RR = 1.00; 95% CI: 0.80–1.24; *p* = 1.00; I^2^ = 0%; Figure 2) [12, 13, 19, 24].

##### 3.2.1.2. Unfavorable GOS Scores

Unfavorable neurological outcomes were defined as a *GOS score of ≤ 3* (i.e., severe disability, vegetative state, or death). The primary time point for assessment was *6 months post-injury*, in line with reporting standards across most included studies.

**Figure 3:**
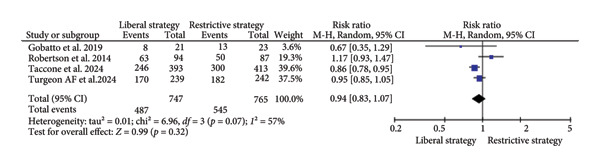
The impact of different transfusion strategies on unfavorable Glasgow Outcome Scale (GOS) scores.

A total of five studies (*n* = 1512) assessed 6‐month unfavorable GOS scores. The results revealed no significant difference between the restrictive and liberal strategy groups (RR = 0.94; 95% CI: 0.83–1.07; *p* = 0.32; I^2^ = 57%; Figure 3) [13, 15, 19, 24]. A sensitivity analysis was conducted, which revealed a statistically significant reduction in unfavorable GOS scores with the restrictive strategy compared to the liberal strategy group (RR = 0.90; 95% CI: 0.82–0.97; *p* = 0.009; I^2^ = 15%; Supplementary Figure 2).

##### 3.2.1.3. RBC Units Transfused per Patient

**Figure 4:**
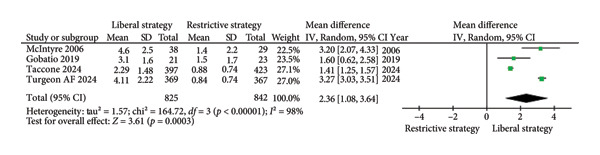
The impact of different transfusion strategies on RBC units transfused per patient.

A total of four studies (*n* = 1667) reported the number of RBC units transfused per patient. The results were statistically significant, with the restrictive strategy group receiving fewer RBC units than the liberal strategy group (MD = 2.36; 95% CI: 1.08 to 3.64; *p* = 0.0003; I^2^ = 98%; Figure 4) [12, 15, 19, 24].

##### 3.2.1.4. Blood Transfusions in ICU (Transfused and Infected Patients)

**Figure 5:**
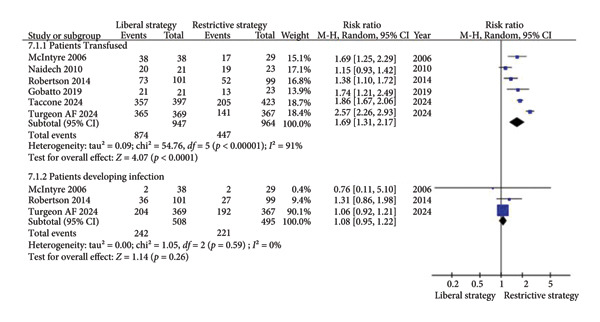
The impact of different transfusion strategies on blood transfusions in ICU patients and the incidence of infection.

A total of six studies (*n* = 1911) reported on blood transfusions in ICU patients. The results revealed a statistically significant higher rate of transfusions in the liberal strategy than in the restrictive strategy group (RR = 1.69; 95% CI: 1.31 to 2.17; *p*  <  0.0001; I^2^ = 91%; Figure 5) [12, 13, 15, 19, 24, 25]. Additionally, four studies (*n* = 1495) evaluated the incidence of infection in ICU patients. No significant difference was found between the restrictive and liberal strategy groups (RR = 1.08; 95% CI: 0.95–1.22; *p* = 0.26; I^2^ = 0%; Figure 5) [12, 13, 19]. Some studies contributed data to both outcomes: RBC units transfused (Figure 4) and number of patients transfused or developing infections (Figure 5), reflecting different aspects of transfusion burden.

##### 3.2.1.5. Length of Stay (ICU and Hospital)

**Figure 6:**
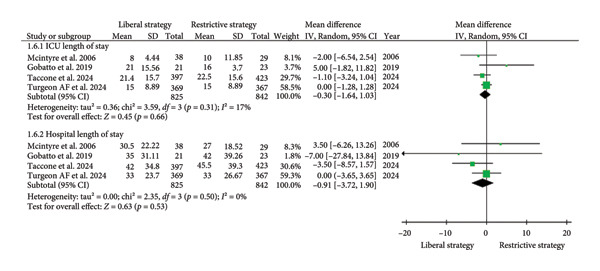
The impact of different transfusion strategies on length of stay in ICU and hospital.

The four studies (*n* = 1667) included in the analysis found no significant difference between the liberal and restrictive strategy groups in terms of ICU length of stay (MD = −0.30; 95% CI: −1.64 to 1.03; *p* = 0.66; I^2^ = 17%; Figure 6) or hospital length of stay (MD = −0.91; 95% CI: −3.72 to 1.03; *p* = 0.53; I^2^ = 0%; Figure 6) [12, 15, 19, 24].

#### 3.2.2. Secondary Outcomes

##### 3.2.2.1. Neurological Adverse Events

A total of four studies (*n* = 1108) assessed the incidence of stroke, showing no significant difference between the liberal and restrictive strategy groups (RR = 0.70; 95% CI: 0.49–1.00; *p* = 0.05; I^2^ = 0%; Supplementary Figure 3) [13, 15, 24, 25]. Similarly, two studies (*n* = 1020) evaluated the incidence of brain hypoxia, with no significant difference found between the two groups (RR = 0.89; 95% CI: 0.56–1.41; *p* = 0.62; I^2^ = 4%; Supplementary Figure 4) [13, 15].

There were two studies (*n* = 88) that reported the incidence of vasospasm, revealing no significant difference between the liberal and restrictive strategy groups (RR = 0.55; 95% CI: 0.15–2.00; *p* = 0.36; I^2^ = 69%; Supplementary Figure 5) [24, 25]. In addition, two studies (*n* = 244) evaluated the incidence of intracranial hypertension requiring therapy and seizures [13, 24]. No significant difference was observed between the groups for intracranial hypertension (RR = 0.99; 95% CI: 0.80–1.22; *p* = 0.90; I^2^ = 41%; Supplementary Figure 6) nor for seizures (RR = 1.41; 95% CI: 0.46–4.32; *p* = 0.55; I^2^ = 0%; Supplementary Figure 7).

Lastly, three studies (*n* = 980) assessed the incidence of ventriculitis, meningitis, or brain abscess, finding no significant difference between the liberal and restrictive strategy groups (RR = 0.84; 95% CI: 0.44–1.62; *p* = 0.60; I^2^ = 0%; Supplementary Figure 8) [13, 19, 24].

##### 3.2.2.2. Non‐Neurological Adverse Events

A total of three studies (*n* = 1064) assessed thromboembolic events and acute myocardial infarction (MI) for the liberal and restrictive strategy groups [13, 15, 24]. The results revealed no significant differences between the groups for thromboembolic events (RR = 1.91; 95% CI: 0.89–4.09; *p* = 0.09; I^2^ = 45%; Supplementary Figure 9) and for MI (RR = 0.64; 95% CI: 0.08–5.13; *p* = 0.67; I^2^ = 0%; Supplementary Figure 10). The four studies (*n* = 1108) evaluating hypotension and ARDS also showed no significant differences between the groups for either outcome (RR = 0.99; 95% CI: 0.82–1.18; *p* = 0.88; I^2^ = 0%; Supplementary Figure 11) for hypotension and (RR = 0.97; 95% CI: 0.58–1.61; *p* = 0.90; I^2^ = 37%; Supplementary Figure 12) for ARDS [13, 15, 24, 25].

Regarding sepsis, four studies (*n* = 1800) showed a statistically significant lower incidence in the restrictive strategy group than in the liberal strategy group (RR = 0.73; 95% CI: 0.56–0.96; *p* = 0.02; I^2^ = 0%; Supplementary Figure 13) [13, 15, 19, 24]. However, two studies (*n* = 936) on bacteremia found no significant difference (RR = 0.89; 95% CI: 0.53–1.49; *p* = 0.66; I^2^ = 0%; Supplementary Figure 14) [13, 19].

For pneumonia, four studies (*n* = 1024) found no significant difference between the liberal and restrictive strategy groups (RR = 0.65; 95% CI: 0.21–2.01; *p* = 0.45; I^2^ = 89%; Supplementary Figure 15) [13, 19, 24, 25]. A sensitivity analysis was also performed; however, the results remained nonsignificant (RR = 1.07; 95% CI: 0.58–1.98; *p* = 0.82; I^2^ = 21%; Supplementary Figure 16).

Finally, two studies (*n* = 244) assessed the incidence of pulmonary embolism (PE) and urinary tract infections (UTIs) [13, 24]. The results showed no significant difference between the groups for PE (RR = 3.13; 95% CI: 0.60–16.44; *p* = 0.18; I^2^ = 0%; Supplementary Figure 17) and UTIs (RR = 1.68; 95% CI: 0.21–13.10; *p* = 0.62; I^2^ = 51%; Supplementary Figure 18).

## 4. Discussion

This analysis systematically evaluates RBC transfusion strategies in neurocritical care, comparing the outcomes of liberal and restrictive transfusion thresholds. By examining mortality, neurological outcomes, adverse events, and significant complications, this research aims to address critical gaps in evidence regarding the optimal transfusion strategy in this vulnerable population. Our study provides a comprehensive analysis pooling data from seven RCTs investigating transfusion strategies [12, 13, 15, 19, 24–26]. The trials enrolled 1941 patients, with 977 in the liberal strategy group and 964 in the restrictive group. With the largest sample size, the Taccone study randomized 850 patients into liberal and restrictive transfusion strategies [15].

The central observation was that there were no significant differences between the restrictive and liberal strategy groups in mortality rates, unfavorable GOS scores, length of stay, and neurological and non‐neurological complications except for sepsis. These findings indicate that restrictive transfusion strategies do not compromise survival or neurological recovery in neurocritical care patients.

The lack of significant differences in outcomes, such as mortality or unfavorable GOS scores, may be attributed to variations in study populations. Differences in baseline characteristics, such as illness severity and comorbidities, can influence results. For example, Turgeon et al. [19] focused on patients with anemia and a population with more severe TBI, which led to an unexpectedly high baseline risk of poor outcomes. As baseline risk increases, detecting minor treatment effects becomes more challenging. Furthermore, study design limitations, such as small sample sizes and inconsistencies in defining restrictive versus liberal strategies, may have reduced statistical power and contributed to overlapping practices.

For instance, in Turgeon et al.’s study [19], the median Hb level in the ICU was 10.8 g/dL in the liberal strategy group and 8.8 g/dL in the restrictive strategy group. Additionally, there was no recommendation to screen for venous thromboembolic events, potentially leading to underreporting of their actual incidence. Some patients may have also received blood transfusions before randomization, which could have reduced differences in Hb levels and transfusion exposure between groups, as noted in Taccone et al.’s study [15]. The small sample size in Gobatto’s study may have also limited the ability to detect subtle differences between groups [24].

We also found that specific outcomes showed no significant differences due to varying injury mechanisms in neurocritical patients, which led to different responses to transfusion strategies. For example, TBI causes direct mechanical damage to the brain, leading to localized injuries and secondary effects such as inflammation and elevated intracranial pressure [27]. In contrast, ischemic stroke results from restricted blood flow to a specific region, causing oxygen deprivation and tissue death [28]. Despite low heterogeneity across studies, indicating some consistency in the observed benefits of restrictive strategies, these findings do not suggest universal advantages across all outcomes.

It is essential to recognize that confounding variables, such as concurrent treatments and variations in ICU management, influence outcomes observed in studies comparing transfusion strategies. Differences in preoperative practices also shape mortality, thromboembolic events, blood loss, and transfusion requirements [29]. Implementing comprehensive patient blood management has been shown to improve clinical outcomes and reduce transfusion utilization, highlighting the significant impact of institutional practices on patient outcomes [30]. Clinicians should carefully consider individual patient factors when determining transfusion thresholds, and future research should focus on standardizing definitions and addressing confounders to understand better the long‐term benefits of restrictive transfusion strategies across diverse populations.

The findings from this meta‐analysis corroborate previous studies, such as Herbert et al. [31], which found that restrictive transfusion thresholds were equally effective as liberal thresholds in ICU patients. Both studies observed no significant differences in mortality or neurological outcomes between the two approaches. Similarly, the results from Yuan et al. [32] supported this view, with no observed effect of restrictive transfusion strategies on mortality (RR 1.00, 95% CI 0.80–1.24). Additionally, this analysis revealed that the liberal transfusion strategy led to a higher number of RBC units transfused per patient (MD: 2.36, 95% CI: 1.08–3.64), a result consistent with findings from Holst et al. [33] and Yuan et al. [32]. Specifically, our study stands out in terms of sample size and scope, incorporating data from seven RCTs and a total of 1941 patients, whereas Yuan et al.’s study [32] was based on just five RCTs and 1528 patients. This produces an extensive and comprehensive dataset that enhances the reliability and generalizability of our conclusions.

Our findings align with those of Tsai et al. [34], who conducted a meta‐analysis with trial sequential analysis of six RCTs and similarly reported no significant differences in unfavorable neurological outcomes or mortality between liberal and restrictive transfusion strategies in acute brain injury patients [34]. Tsai et al. [34] emphasized that restrictive thresholds of 7–8 g/dL appeared safe, avoiding unnecessary transfusions, which is consistent with our conclusion that restrictive transfusion does not worsen outcomes. In contrast, Nguyen et al. [35] analyzed five RCTs and suggested that liberal transfusion strategies may reduce the risk of sepsis and improve 6‐month neurological recovery compared with restrictive thresholds [35]. These differing results reflect variations in trial inclusion and outcome definitions, underscoring ongoing uncertainty and the need for further high‐quality RCTs.

Zhang et al. [36] reached a different conclusion, suggesting that restrictive transfusion strategies may lower in‐hospital mortality in critically ill patients with anemia. However, no such difference in ICU or hospital mortality was found in this meta‐analysis. This contrast may stem from differences in the timing of the studies, as our analysis includes more recent trials, such as those by Turgeon et al. [19] and Taccone et al. [15], which could explain divergent results. Moreover, our meta‐analysis considered a wide range of primary and secondary outcomes, offering a more robust and detailed analysis than Zhang et al.’s study.

After addressing the primary objectives, we evaluated secondary complications. Across all studies in the meta‐analysis, the liberal transfusion strategy consistently resulted in significantly higher transfusion rates than the restrictive strategy. Blood transfusions during ICU admissions were statistically significant, with the liberal strategy leading to considerably more transfusions. However, no significant association was found between the transfusion strategy and the risk of infections. Restrictive transfusion strategies may help reduce complications such as sepsis, likely due to decreased exposure to blood products. This lowers the risk of immune modulation and its associated adverse effects. Research suggests that restrictive transfusion strategies reduce the risk of venous thromboembolic events compared to liberal strategies by minimizing unnecessary transfusions and decreasing blood viscosity and hypercoagulability [37]. Furthermore, stored blood products can accumulate pro‐inflammatory cytokines or undergo structural changes that contribute to complications. By minimizing unnecessary transfusions, these risks are reduced. For instance, cytokines, chemokines, and other immunomodulatory molecules in stored platelet concentrations can vary in their secretion over time during storage and may modulate the recipient’s immune system upon transfusion [38].

Another important takeaway from this analysis is the considerable heterogeneity in transfusion rates across the studies, which reflects substantial differences in clinical practices, patient populations, and decision‐making processes. This variability has often been overlooked in past research, but understanding these differences is crucial for refining transfusion protocols in neurocritical care settings. Our study also evaluated key complications, including stroke and pneumonia, which may impact recovery trajectories. In contrast, no significant association was found between transfusion strategy and the risk of stroke, PE, or ARDS. These results lend further support to the idea that restrictive transfusion strategies may not exacerbate these major complications and may be equally or even more beneficial than liberal strategies for critically ill patients.

In the sensitivity analysis for unfavorable GOS scores and pneumonia, Turgeon et al. [19] and Robertson et al. [13] were excluded, respectively. Excluding these studies helped reduce heterogeneity, suggesting that the remaining studies were more consistent and improved the pooled results’ reliability. In Turgeon et al.’s study [19], the high heterogeneity was likely due to the median Hb levels in the ICU, with 10.8 g/dL for the liberal strategy and 8.8 g/dL for the restrictive strategy. In Robertson et al. [13], the high variability likely arose from the belief that maintaining adequate oxygenation to the injured brain was critical for TBI patients, with the study powered to detect a significant difference in outcomes based on the transfusion threshold.

We attempted to perform sensitivity analyses for outcomes with higher heterogeneity but could not do so due to significant variability across the studies. This limitation arose from methodological inconsistencies and data constraints. Although sensitivity analyses were conducted for unfavorable GOS and pneumonia, the inability to analyze other outcomes more thoroughly diminishes confidence in the results. It may overlook biases or confounding factors, reducing the overall reliability of the study’s conclusions.

Substantial heterogeneity was observed in blood transfusion outcomes, particularly in the transfusion of RBC units and total blood transfusions in ICU patients. Moderate to significant heterogeneity was also noted for vasospasm and pneumonia. In contrast, low heterogeneity was found in several primary and secondary outcomes, including mortality rates at various time points (ICU, hospital, 30‐day, 6‐month, and long‐term) and length of ICU or hospital stay, where results were consistent across studies. Similarly, the incidence of infection, unfavorable GOS scores, and most neurological adverse events, such as stroke, brain hypoxia, and seizures, exhibited low heterogeneity. Non‐neurological complications like hypotension, ARDS, sepsis, and bacteremia also showed low to moderate heterogeneity, suggesting consistent findings across studies.

Regarding neurological outcomes, this meta‐analysis found no significant differences between restrictive and liberal transfusion strategies in neurocritical patients. This suggests that RBC transfusions may not significantly affect neurological recovery or complications in these cases. It contrasts with previous research, such as Taccone et al. [15], which observed significantly fewer ischemic brain events in patients assigned to the liberal transfusion group. The variability in responses to transfusion strategies among different types of neurological injuries (ischemic stroke, TBI, or SAH) should be considered. Although this analysis focused on neurocritical patients, we also observed that in septic patients, a restrictive transfusion strategy reduced the risk of complications by 27% compared to the liberal strategy, highlighting the variability of transfusion strategy outcomes across different patient populations.

### 4.1. Clinical Implications and Future Prospects

Restrictive transfusion strategies effectively reduce transfusion‐related complications, such as sepsis, without adversely affecting critical outcomes like mortality or neurological recovery. By reducing unnecessary exposure to blood products, these strategies lower the risk of immune modulation and side effects from stored blood, like proinflammatory cytokines. While restrictive strategies are preferred to minimize transfusion risks, a more liberal approach may be better in cases like acute MI and anemia. The Myocardial Ischemia and Transfusion (MINT) trial explored this by comparing liberal and restrictive transfusion strategies in MI patients. While the liberal strategy did not significantly reduce the risk of MI or 30‐day mortality, it could not conclusively rule out potential harms associated with the restrictive approach. These findings suggest that in high‐risk populations, such as those with significant cardiovascular disease, a more liberal transfusion strategy may be necessary to prevent adverse ischemic events. Therefore, an individualized assessment of patient risk factors and clinical status is essential when determining the most appropriate transfusion strategy [3].

In addition to TBI and ischemic stroke, SAH represents a distinct neurocritical condition in which transfusion thresholds may warrant different targets. SAH refers to bleeding into the space between the pia mater and the arachnoid membrane of the central nervous system [39]. Approximately 85% of SAH cases result from ruptured aneurysms, around 10% from non‐aneurysmal perimesencephalic hemorrhage, which typically carries a favorable prognosis, and the remaining 5% from other uncommon causes [40]. The pathogenesis of SAH, characterized by cerebral vasospasm, delayed cerebral ischemia, and impaired autoregulation, can alter cerebral oxygen delivery and metabolic demands, raising the possibility that avoiding even mild anemia could be beneficial in this subgroup [41–43]. SAH is distinct from both TBI and ischemic stroke in terms of its underlying cause and secondary injury pathways. Whereas TBI arises from direct mechanical trauma and ischemic stroke from vascular occlusion with focal hypoperfusion, SAH involves bleeding into the subarachnoid space, most commonly due to aneurysmal rupture [41]. Given this distinct pathophysiology, transfusion thresholds in SAH may need to be individualized, as maintaining optimal Hb levels could play a greater role in preventing vasospasm and delayed cerebral ischemia. In a preplanned secondary analysis of the TRAIN trial, outcomes were evaluated among SAH patients randomized to a liberal RBC transfusion threshold (< 9 g/dL) or a restrictive threshold (< 7 g/dL). Multivariable analysis demonstrated that assignment to the liberal group was independently associated with a higher likelihood of favorable neurological recovery at 180 days, defined by a GOS‐Extended score of 6–8. Notably, patients in the liberal transfusion arm also experienced a lower incidence of cerebral ischemia from any cause [44]. These findings highlight the need for SAH‐specific transfusion strategies, as uniform thresholds across neurocritical populations may not adequately address the unique pathophysiological challenges of this condition.

Despite the benefits of restrictive strategies, several knowledge gaps remain. For instance, optimal thresholds for restrictive versus liberal transfusions are not clearly defined, and there is a lack of long‐term data to guide decision‐making for specific subgroups, such as trauma patients or those with pre‐existing comorbidities. Trauma patients often require tailored transfusion protocols due to acute hemorrhage and coagulopathy. Studies suggest that restrictive transfusion triggers are beneficial in trauma settings, but the optimal Hb thresholds remain undetermined [45]. Similarly, while restrictive strategies may be safe for many patients with chronic cardiovascular disease, some individuals may have varying tolerance to anemia, requiring caution in cases involving unstable angina or acute MI [31].

Research focused on standardizing transfusion practices and exploring subgroup‐specific outcomes is essential to address these gaps. The long‐term implications of restrictive transfusion strategies need further investigation. These findings are critical for refining ICU guidelines and supporting restrictive strategies as a safer default approach. Uniform Hb concentration thresholds should be established to guide transfusion decisions. Transfusion should be considered when Hb levels fall below 7 g/dL for hemodynamically stable hospitalized adults. For patients with pre‐existing cardiovascular conditions or those undergoing cardiac or orthopedic surgery, a threshold of 7.5–8 g/dL should be applied. To optimize transfusion thresholds and strategies, studies focusing on diverse patient populations, including those with oncological or hematologic disorders, should be prioritized [3].

Institutional protocols that minimize transfusion requirements, such as optimizing erythropoiesis and reducing blood loss, should be implemented and tailored to specific patient populations and clinical scenarios [46]. Additionally, a framework for the periodic review and revision of transfusion guidelines should be established to ensure the incorporation of the latest research findings and to keep recommendations current and evidence‐based [47]. Therefore, individualized decision‐making is the key to balancing the risks and benefits of transfusion in patient outcomes. Refining guidelines in critical care requires enhancing evidence‐based thresholds and incorporating subgroup‐specific recommendations.

### 4.2. Limitations

Our study acknowledges several limitations that may affect the interpretation and generalizability of the results. First, significant heterogeneity was observed in some analyses, particularly concerning transfusion rates and unfavorable GOS. This variability may affect the reliability and consistency of the results. Heterogeneity across studies may stem from variations in study design, intervention types, sample populations, and follow‐up durations. Differences in participant characteristics, such as age, sex, underlying conditions, and the specific need for transfusion (e.g., TBI, ICU patients, or surgical cases), also contribute to variability. The seven RCTs evaluating transfusion strategies in neurologically injured patients differ greatly in their participant demographics and inclusion criteria, especially regarding the type of injury, timing, and severity scores of neurological conditions. The study by Turgeon et al. involved adults (≥ 18 years) experiencing moderate to severe TBI, characterized by a Glasgow Coma Scale (GCS) score of 3–12 and anemia (Hb ≤ 10 g/dL), while excluding individuals who received transfusions after ICU admission but prior to randomization. The study by Taccone et al. expanded its patient criteria to those with TBI, SAH, or ICH, having a GCS ≤ 13, Hb ≤ 9 g/dL, and a forecasted ICU admission of ≥ 3 days, launching the protocol within 24 h after meeting the criteria. Gobatto et al. examined patients with moderate to severe TBI (GCS ≤ 12), Hb < 9 g/dL, and enforced strict exclusions for bilateral fixed pupils and prior neurological impairments. Robertson et al. enrolled patients with closed head injury, unresponsive following resuscitation, and accepted within 6 h postinjury, excluding those with a GCS of three and injuries deemed nonsurvivable. Naidech et al. focused on aneurysmal SAH patients, utilizing the World Federation of Neurological Surgeons (WFNS) scale, including grades 2–4 or grade 1 with significant SAH in imaging, and mandated that aneurysm obliteration be either completed or imminent; patients with initial CT infarcts were excluded. The study by McIntyre et al. conducted a retrospective analysis of a subgroup of trauma patients suffering from closed head injuries who were initially enrolled with Hb levels ≤ 9 g/dL and admitted to the ICU within 72 h, with illness severity evaluated through GCS, Injury Severity Score (ISS), and Multiple Organ Dysfunction (MOD) scores.

The differences in defining liberal and restrictive transfusion strategies among the RCTs lead to considerable heterogeneity, impacting the interpretation and comparability of their outcomes. In the study by Turgeon et al., the liberal approach involved starting transfusions at a Hb level of ≤ 10 g/dL, whereas the restrictive approach applied a threshold of ≤ 7 g/dL. Similarly, Taccone et al. categorized liberal and restrictive strategies as < 9 g/dL and < 7 g/dL, respectively. Gobatto et al. used the identical thresholds as Taccone, upholding a steady yet not universal definition. McIntyre et al. used a liberal strategy starting at 10.0 g/dL and a restrictive one at 7.0 g/dL, but with well‐defined maintenance parameters. Robertson et al. evaluated the impact of transfusing at levels exceeding 10 g/dL, indicating a liberal‐only threshold without directly comparing it to a restrictive alternative. These variations in operational definitions affect the timing of transfusions, the amount of RBCs given, and the patient’s exposure to risks associated with transfusions.

The differences in transfusion thresholds lead to clinical variation in managing patients, influencing results such as brain oxygen levels, risk of vasospasm, and death rates. This lack of consistency further hinders attempts to combine results from different studies or create cohesive clinical guidelines. Thus, while each trial provides valuable insights, the differing definitions of “liberal” and “restrictive” strategies highlight the need for standardized protocols in future neurocritical care transfusion research.

The seven RCTs displayed notable differences in study design, contributing to methodological heterogeneity. Larger, multicenter trials such as those by Turgeon and Taccone adopted broader inclusion criteria and pragmatic approaches, with a larger population size, whereas smaller, single‐center studies like those by Zygun and Naidech focused on specific populations and involved detailed physiological monitoring. As an example, Zygun et al. used intensive brain tissue oxygenation monitoring in the population as an intervention. Protocol durations also differed, as Gobatto et al. maintained the transfusion strategy for 14 days or until ICU discharge, while Taccone applied it over a 28‐day period. Timing of enrollment varied widely too, as Robertson et al. enrolled patients within 6 h postinjury, targeting the hyperacute phase, whereas Taccone et al. allowed enrollment up to 10 days postinjury, potentially including patients at very different clinical stages. These design differences complicate direct comparisons and may increase the heterogeneity across the studies further. Moreover, screening for various thromboembolic events was not advised in several included studies, including Turgeon et al. Potentially leading to underreporting of the true incidence of these events. This methodological limitation could have influenced the reported complication rates and contributes to the variability observed across studies. Additionally, discrepancies in defining “liberal” (Hb > 9 g/dL) versus “restrictive” (Hb < 7 g/dL) transfusion strategies, along with differences in healthcare settings (e.g., ICU protocols and resources), further contribute to observed heterogeneity. Other factors, such as variations in measurement tools, statistical methods, and data collection techniques, can also introduce variability. Many of the included studies were single‐center trials that may not represent more significant, more diverse populations, so the generalizability of the results is limited by the patient groups and specific settings studied.

Furthermore, potential publication bias and incomplete data for some outcomes may have affected the completeness of the analysis; if studies with less observed effects on specific outcomes (e.g., transfusion rates or GOS scores) were less likely to be published, the analysis could be biased toward positive results. Therefore, the study results may not accurately reflect the accurate distribution of outcomes across all relevant studies, leading to overestimating treatment effects or consequences.

Sensitivity analyses were also attempted for outcomes with high heterogeneity, such as the number of RBC units transfused. However, due to significant methodological variability and lack of consistent reporting across the included trials, these analyses did not result in meaningful reductions in I^2^ and were therefore not reported. This reflects inherent differences in study designs, transfusion thresholds, and patient populations rather than shortcomings in analysis and must be considered when interpreting these findings.

Some deviations from the registered PROSPERO protocol should be acknowledged. While the protocol permitted inclusion of both randomized and nonrandomized studies, we restricted the analysis to RCTs to enhance methodological rigor and minimize bias. Additionally, although mortality and unfavorable GOS outcomes were designated as the primary outcomes in the PROSPERO registration, we also analyzed additional neurological and non‐neurological outcomes as secondary or exploratory outcomes. These additions were intended to provide a more comprehensive synthesis of available evidence but should be interpreted in light of their exploratory nature. These factors should be considered when interpreting this study’s conclusions.

## 5. Conclusion

In conclusion, the comparison between restrictive and liberal transfusion strategies shows that restrictive approaches are as practical as liberal ones in terms of mortality, neurological outcomes, and most major non‐neurological complications. Restrictive strategies are associated with fewer blood transfusions and a lower incidence of sepsis without significantly affecting outcomes like mortality, GOS, or complications such as pneumonia, PE, and ARDS. The potential benefits of restrictive strategies, such as reduced exposure to blood products and a decreased risk of complications like sepsis, suggest that restrictive transfusion should be considered a safer default approach. However, the heterogeneity observed across studies due to variations in study design, patient populations, and transfusion threshold definitions highlights the importance of individualized decision‐making, particularly for subgroups with diverse neurological conditions or comorbidities. This variability underscores the need for standardized and more refined ICU guidelines to balance risks and benefits better. While the evidence strongly supports restrictive transfusion strategies, there are notable research gaps, particularly regarding long‐term outcomes, subgroup‐specific effects, and the inconsistency of transfusion thresholds. Future studies should address these gaps, focusing on long‐term impacts and developing evidence‐based, subgroup‐specific recommendations. Despite the promising results, it is important to consider the limitations, including study heterogeneity, potential publication bias, and variability in transfusion thresholds, when interpreting these findings.

**Nomenclature**
TBI: Traumatic brain injury
SAH: Subarachnoid hemorrhage
ICH: Intracerebral hemorrhage
Hb: Hemoglobin
ARDS: Acute respiratory distress syndrome
RBC: Red blood cell
ICU: Intensive care unit
RCT: Randomized Controlled Trial
GOS: Glasgow Outcome Scale
MINT: Myocardial Ischemia and Transfusion
PE: Pulmonary embolism
UTI: Urinary tract infection
MI: Myocardial infarction

## Ethics Statement

The authors have nothing to report.

## Consent

The authors have nothing to report.

## Conflicts of Interest

The authors declare no conflicts of interest.

## Author Contributions

Ayesha Shaukat: conceptualization, discussion, writing–review and editing, project administration. Muhammad Ahmed Zahoor: formal analysis, data curation, writing–original draft. Komal Khan: writing–original draft, writing–reviewing and editing. Aiman Shahid Khan: formal analysis, data curation. Rubaisha Saleem: formal analysis, data curation. Anupama Ariyasiri: writing–original draft. Syed Abdul Aziz Jameel: writing–original draft, writing–reviewing and editing. Shahab Afridi: writing–original draft, writing–reviewing and editing. Syeda Javeria Salman: methodology, writing–reviewing and editing. Noor Naeem: writing–original draft. Marib Ashraf: writing–original draft. Amamah Rauf Chaudhry: writing–original draft. Zobia Ahmad: writing–reviewing and editing. Muhammad Omar Larik: writing–reviewing and editing. Muhammad Hasanain: writing–reviewing and editing. Muhammad Umair Anjum: writing–reviewing and editing. Aymar Akilimali: writing–reviewing and editing.

## Funding

The authors received no specific funding for this work.

## Supporting Information

Supplementary Table 1: Detailed search strategy used for each database (PubMed, Cochrane, ScienceDirect, Google Scholar) in the systematic review.

Supplementary Table 2: Quality of evidence for all outcomes using the GRADE (Grading of Recommendations Assessment, Development, and Evaluation) framework.

Supplementary Figure 1: Risk of bias (A) and traffic light plot (B) for the included trials.

Supplementary Figure 2: Sensitivity analysis showing the impact of transfusion strategies on unfavorable Glasgow Outcome Scale (GOS) scores.

Supplementary Figure 3: Impact of transfusion strategies on stroke.

Supplementary Figure 4: Impact on brain hypoxia.

Supplementary Figure 5: Impact on vasospasm.

Supplementary Figure 6: Impact on intracranial hypertension requiring therapy.

Supplementary Figure 7: Impact on seizures.

Supplementary Figure 8: Impact on ventriculitis, meningitis, or brain abscess.

Supplementary Figure 9: Impact on thromboembolic events.

Supplementary Figure 10: Impact on acute myocardial infarction (MI).

Supplementary Figure 11: Impact on hypotension.

Supplementary Figure 12: Impact on acute respiratory distress syndrome (ARDS).

Supplementary Figure 13: Impact on sepsis.

Supplementary Figure 14: Impact on bacteremia.

Supplementary Figure 15: Impact on pneumonia.

Supplementary Figure 16: Sensitivity analysis showing the impact on pneumonia.

Supplementary Figure 17: Impact on pulmonary embolism (PE).

Supplementary Figure 18: Impact on urinary tract infections (UTIs).

## Supporting information


**Supporting Information** Additional supporting information can be found online in the Supporting Information section.

## Data Availability

The data that support the findings of this study are available in the supporting information of this article.
