# Supplementary material for: Liberal Versus Restrictive Blood Transfusion Strategies in Neurocritical Care: A Systematic Review and Meta‐Analysis of Randomized Controlled Trials
Source: Crit Care Res Pract. 2026 Jan 12;2026:6179847. doi: 10.1155/ccrp/6179847 (PMC12793887; doi:10.1155/ccrp/6179847)
Supplement: Supplementary file 1 — Supporting Information Additional supporting information can be found online in the Supporting Information section. [file CCRP-2026-6179847-s001.docx]

**SUPPLEMENTARY FILE**

**Liberal vs. Restrictive Blood Transfusion Strategies in Neurocritical Care: A Systematic Review and Meta-Analysis of Randomized Controlled Trials**

**Authors:**

Ayesha Shaukat, MBBS^1^, Muhammad Ahmed Zahoor, MBBS^1^, Komal Khan, MBBS^2^, Aiman Shahid Khan, MBBS^1^, Rubaisha Saleem, MBBS^1^, Anupama Ariyasiri, MBBS^3^, Syed Abdul Aziz Jameel, MBBS^3^, Shahab Afridi, MBBS^4^, Syeda Javeria Salman, MBBS^1^, Noor Naeem, MBBS^5^, Marib Ashraf, MBBS^5^, Amamah Rauf Chaudhry, MBBS^5^, Zobia Ahmad, MBBS^1^, Muhammad Omar Larik, MD^3^, Muhammad Hasanain, MD^1^, Muhammad Umair Anjum, MD^6^, Aymar Akilimali, MD^7^

**Affiliations:**

**^1^** Department of Medicine, Dow Medical College, Karachi, Pakistan

**^2^** Department of Medicine, Ziauddin University, Karachi, Pakistan

**^3^** Department of Medicine, Dow International Medical College, Karachi, Pakistan

**^4^** Department of Medicine, Ayub Medical College, Abbottabad, Pakistan

**^5^** Department of Medicine, Rashid Latif Medical College, Lahore, Pakistan

**^6^** Department of Medicine, Mayo Clinic, Phoenix, Arizona, United States

**^7^** Department of Research, Medical Research Circle, Goma, Democratic Republic of the Congo

**Article Category:** Systematic Review and Meta-Analysis

**Corresponding author**: **Aymar Akilimali**

**Address**: Medical Research Circle, Postal code 73 Gisenyi, Goma, DR Congo

**Email**: [aymarakilimali@gmail.com](mailto:aymarakilimali@gmail.com) (**0000-0001-9393-1215**)

| **Database** | **Search Strategy** | **Number of Studies** |
| --- | --- | --- |
| PubMed | "Traumatic Brain Injury"[MeSH] OR "traumatic brain injury"[All Fields] OR TBI[All Fields] OR "head injury"[All Fields] | 3280 |
|  | "Traumatic Brain Injury"[MeSH] OR "traumatic brain injury"[All Fields] OR TBI[All Fields] OR "head injury"[All Fields] AND "Subarachnoid Hemorrhage"[MeSH] OR "subarachnoid hemorrhage"[All Fields] OR SAH[All Fields] | 106 |
|  | "Traumatic Brain Injury"[MeSH] OR "traumatic brain injury"[All Fields] OR TBI[All Fields] OR "head injury"[All Fields] AND "Subarachnoid Hemorrhage"[MeSH] OR "subarachnoid hemorrhage"[All Fields] OR SAH[All Fields] AND "Intracerebral Hemorrhage"[MeSH] OR "intracerebral hemorrhage"[All Fields] OR ICH[All Fields] | 22 |
|  | ((("Traumatic Brain Injury"[MeSH] OR "traumatic brain injury"[All Fields] OR TBI[All Fields] OR "head injury"[All Fields]) AND ("Subarachnoid Hemorrhage"[MeSH] OR "subarachnoid hemorrhage"[All Fields] OR SAH[All Fields])) AND ("Intracerebral Hemorrhage"[MeSH] OR "intracerebral hemorrhage"[All Fields] OR ICH[All Fields])) AND ("Anemia"[MeSH] OR anemia[All Fields] OR "low hemoglobin"[All Fields]) | 2 |
|  | ((("Traumatic Brain Injury"[MeSH] OR "traumatic brain injury"[All Fields] OR TBI[All Fields] OR "head injury"[All Fields]) AND ("Subarachnoid Hemorrhage"[MeSH] OR "subarachnoid hemorrhage"[All Fields] OR SAH[All Fields])) AND ("Intracerebral Hemorrhage"[MeSH] OR "intracerebral hemorrhage"[All Fields] OR ICH[All Fields])) AND ("Anemia"[MeSH] OR anemia[All Fields] OR "low hemoglobin"[All Fields]) AND "Blood Transfusion"[MeSH] OR "red blood cell transfusion"[All Fields] OR "transfusion threshold"[All Fields] OR "restrictive transfusion"[All Fields] OR "liberal transfusion"[All Fields] | 588 |
| Cochrane | anemia | 27553 |
|  | (anemia) AND (traumatic brain injury OR subarachnoid hemorrhage OR intracerebral hemorrhage OR neurocritical care) | 11751 |
|  | (anemia) AND (traumatic brain injury OR subarachnoid hemorrhage OR intracerebral hemorrhage OR neurocritical care) AND (liberal transfusion OR higher hemoglobin threshold ) | 853 |
|  | (anemia) AND (traumatic brain injury OR subarachnoid hemorrhage OR intracerebral hemorrhage OR neurocritical care) AND (liberal transfusion OR higher hemoglobin threshold ) AND (restrictive transfusion OR lower hemoglobin threshold) AND (mortality OR neurological outcome OR Glasgow Outcome Scale OR vasospasm OR stroke OR intracranial hypertension OR deep vein thrombosis OR acute respiratory distress syndrome OR ARDS OR functional independence OR quality of life OR depression OR ICU stay OR hospital stay) | 506482 |
|  | #1 AND #2 AND #3 AND #4 AND #5 | 26 |
| Science Direct | TITLE-ABS-KEY(anemia) | 1141 |
|  | TITLE-ABS-KEY(anemia) AND TITLE-ABS-KEY("traumatic brain injury" OR "subarachnoid hemorrhage" OR "intracerebral hemorrhage") | 98 |
|  | TITLE-ABS-KEY(anemia) AND TITLE-ABS-KEY("traumatic brain injury" OR "subarachnoid hemorrhage" OR "intracerebral hemorrhage") AND TITLE-ABS-KEY("blood transfusion" OR "transfusion threshold" OR "restrictive transfusion" OR "liberal transfusion") | 43 |
|  | TITLE-ABS-KEY(anemia) AND TITLE-ABS-KEY("traumatic brain injury" OR "subarachnoid hemorrhage" OR "intracerebral hemorrhage") AND TITLE-ABS-KEY("blood transfusion" OR "transfusion threshold" OR "restrictive transfusion" OR "liberal transfusion") AND TITLE-ABS-KEY(neurological ourtcome) | 40 |
| Google Scholar | anemia AND (traumatic brain injury OR subarachnoid hemorrhage OR intracerebral hemorrhage OR neurocritical care) AND (liberal transfusion OR higher hemoglobin threshold) AND (restrictive transfusion OR lower hemoglobin threshold) AND (mortality OR neurological outcome OR Glasgow Outcome Scale OR vasospasm OR stroke OR intracranial hypertension OR deep vein thrombosis OR ARDS OR functional independence OR quality of life OR ICU stay OR hospital stay) | 1320 |

**Supplementary Table 1:** Detailed search strategy used for each database.

**Author(s):** Muhammad Ahmed Zahoor, Ayesha Shaukat

**Question:** Liberal Strategy compared to Restrictive Strategy for Neurocritical patients

**Setting:** Neurocritical patients receiving RBC transfusions in the ICU

**Bibliography:** 1,2,3,4,5,6,7

| **Certainty assessment** | | | | | | | **№ of patients** | | **Effect** | | **Certainty** | **Importance** |
| --- | --- | --- | --- | --- | --- | --- | --- | --- | --- | --- | --- | --- |
| **№ of studies** | **Study design** | **Risk of bias** | **Inconsistency** | **Indirectness** | **Imprecision** | **Other considerations** | **Liberal Strategy** | **Restrictive Strategy** | **Relative (95% CI)** | **Absolute (95% CI)** |  |  |

**ICU Mortality**

| 3 | randomised trials | not serious | not serious | not serious | serious^a,b^ | none | 67/428 (15.7%) | 66/419 (15.8%) | **RR 0.74**  (0.28 to 1.91) | **41 fewer per**  **1,000**  (from 113 | ⨁⨁⨁◯  Moderate^a,b^ | CRITICAL |
| --- | --- | --- | --- | --- | --- | --- | --- | --- | --- | --- | --- | --- |
|  |  |  |  |  |  |  |  |  |  | fewer to 143 |  |  |
|  |  |  |  |  |  |  |  |  |  | more) |  |  |

**Hospital Mortality**

| 3 | randomised trials | not serious | not serious | not serious | serious^a,b^ | none | 91/428 (21.3%) | 91/419 (21.7%) | not estimable |  | ⨁⨁⨁◯  Moderate^a,b^ | CRITICAL |
| --- | --- | --- | --- | --- | --- | --- | --- | --- | --- | --- | --- | --- |

**Mortality at 30 days**

| 2 | randomised trials | not serious | not serious | not serious | serious^a^ | none | 87/435 (20.0%) | 99/447 (22.1%) | **RR 0.91**  (0.70 to 1.18) | **20 fewer per**  **1,000**  (from 66 fewer | ⨁⨁⨁◯  Moderate^a^ | CRITICAL |
| --- | --- | --- | --- | --- | --- | --- | --- | --- | --- | --- | --- | --- |
|  |  |  |  |  |  |  |  |  |  | to 40 more) |  |  |

**Mortality at 6 months**

| 3 | randomised trials | not serious | not serious | not serious | serious^a^ | none | 118/491 (24.0%) | 117/487 (24.0%) | **RR 0.98**  (0.67 to 1.44) | **5 fewer per**  **1,000**  (from 79 fewer | ⨁⨁⨁◯  Moderate^a^ | CRITICAL |
| --- | --- | --- | --- | --- | --- | --- | --- | --- | --- | --- | --- | --- |
|  |  |  |  |  |  |  |  |  |  | to 106 more) |  |  |

**Long term mortality**

| 4 | randomised trials | not serious | not serious | not serious | serious^a^ | none | 123/529 (23.3%) | 122/516 (23.6%) | **RR 1.00**  (0.80 to 1.24) | **0 fewer per**  **1,000**  (from 47 fewer | ⨁⨁⨁◯  Moderate^a^ | CRITICAL |
| --- | --- | --- | --- | --- | --- | --- | --- | --- | --- | --- | --- | --- |
|  |  |  |  |  |  |  |  |  |  | to 57 more) |  |  |

**Unfavorable GOS outcomes**

| 4 | randomised trials | not serious | not serious | not serious | serious^a^ | none | 487/747 (65.2%) | 545/765 (71.2%) | **RR 0.94**  (0.83 to 1.07) | **43 fewer per**  **1,000**  (from 121 | ⨁⨁◯◯  Low^a,c^ | CRITICAL |
| --- | --- | --- | --- | --- | --- | --- | --- | --- | --- | --- | --- | --- |
|  |  |  |  |  |  |  |  |  |  | fewer to 50 |  |  |
|  |  |  |  |  |  |  |  |  |  | more) |  |  |

**RBC units per patient**

| 4 | randomised trials | not serious | not serious | not serious | very serious^d^ | none | 825 | 842 | - | MD **2.36**  **higher**  (1.08 higher  to 3.64 higher) | ⨁⨁◯◯  Low^d^ | IMPORTANT |
| --- | --- | --- | --- | --- | --- | --- | --- | --- | --- | --- | --- | --- |

**Patients receiving blood transfusion during ICU**

| 6 | randomised trials | not serious | not serious | not serious | not serious | none | 874/992 (88.1%) | 447/976 (45.8%) | **RR 1.56**  (1.18 to 2.05) | **256 more per**  **1,000**  (from 82 more | ⨁⨁◯◯  Low^d^ | IMPORTANT |
| --- | --- | --- | --- | --- | --- | --- | --- | --- | --- | --- | --- | --- |
|  |  |  |  |  |  |  |  |  |  | to 481 more) |  |  |

**Transfused patients developing infection**

| 3 | randomised trials | not serious | not serious | not serious | serious^a^ | none | 242/508 (47.6%) | 221/495 (44.6%) | **RR 1.08**  (0.95 to 1.22) | **36 more per**  **1,000**  (from 22 fewer | ⨁⨁⨁◯  Moderate^a^ | CRITICAL |
| --- | --- | --- | --- | --- | --- | --- | --- | --- | --- | --- | --- | --- |
|  |  |  |  |  |  |  |  |  |  | to 98 more) |  |  |

**ICU length of stay**

| 4 | randomised trials | not serious | not serious | not serious | serious^a^ | none | 825 | 842 | - | MD **0.3 lower**  (1.64 lower to  1.03 higher) | ⨁⨁⨁◯  Moderate^a^ | CRITICAL |
| --- | --- | --- | --- | --- | --- | --- | --- | --- | --- | --- | --- | --- |

**Hospital length of stay**

| 4 | randomised trials | not serious | not serious | not serious | serious^a^ | none | 825 | 842 | - | MD **0.91 lower**  (3.72 lower to  1.9 higher) | ⨁⨁⨁◯  Moderate^a^ | CRITICAL |
| --- | --- | --- | --- | --- | --- | --- | --- | --- | --- | --- | --- | --- |

**CI:** confidence interval; **MD:** mean difference; **RR:** risk ratio

**Explanations**

1. Confidence Interval crossing both benefit and harm
2. Wider Confidence Interval
3. High heterogeneity which resolved with sensitivity analysis
4. High heterogeneity with no clear explanation

**References**

1., Mc Intyre,et,al. Effect of a liberal versus restrictive transfusion strategy on mortality in patients with moderate to severe head injury.2006. 2., Zygun,et,al. The effect of red blood cell transfusion on cerebral oxygenation and metabolism after severe traumatic brain injury.2009.

3., Naidech,et,al. Prospective, randomiz ed trial of higher goal hemoglobin after subarachnoid hemorrhage.2010.

4., Robertson,et,al. Effect of erythropoietin and transfusion threshold on neurological recovery after traumatic brain injury: a randomiz ed clinical trial.2014. 5., Gobatto,et,al. Transfusion requirements after head trauma: a randomiz ed feasibility controlled trial.2019.

6., Taccone,et,al. Restrictive vs Liberal Transfusion Strategy in Patients With Acute Brain Injury: The TRAIN Randomiz ed Clinical Trial.2024. 7., Turgeon,AF,et,al. Liberal or Restrictive Transfusion Strategy in Patients with Traumatic Brain Injury.2024.

**Supplementary Table 2:** The quality of evidence using the GRADE (Grading of Recommendations Assessment, Development, and Evaluation) framework.

| **Study (year)** | **D1: Randomization process** | **D2: Deviations from intended interventions** | **D3: Missing outcome data** | **D4: Measurement of outcome** | **D5: Selection of reported result** | **Overall** |
| --- | --- | --- | --- | --- | --- | --- |
| Turgeon AF et al., 2024 | Low risk | Low risk | Low risk | Low risk | Low risk | Low risk |
| Taccone et al., 2024 | Low risk | Some concerns | Low risk | Low risk | Low risk | Some concerns^1^ |
| Gobatto et al., 2019 | Low risk | High risk | Low risk | Low risk | High risk | High risk^2^ |
| Robertson et al., 2014 | Low risk | Low risk | Low risk | Low risk | Low risk | Low risk |
| Naidech et al., 2010 | Low risk | Some concerns | Low risk | Low risk | High risk | High risk^3^ |
| Zygun et al., 2009 | Low risk | Low risk | Low risk | Low risk | Low risk | Low risk |
| McIntyre et al., 2006 | Some concerns | Some concerns | Low risk | High risk | High risk | High risk^4^ |

^1^ D2 – Some concerns: Trial was open-label, with limited details on adherence and blinding; deviations from intended intervention could not be fully excluded.

^2^ D2 – High risk: Lack of blinding and possible deviations in perioperative management; **D5 – High risk:** no prespecified analysis plan or trial registration identified, raising concerns of selective reporting.

^3^ **D2 – Some concerns:** Study design was open-label with insufficient information on adherence; **D5 – High risk:** incomplete reporting of prespecified outcomes, raising suspicion of selective reporting.

^4^ **D1 – Some concerns:** Randomization method and allocation concealment not clearly described; **D2 – Some concerns:** unblinded trial with possible deviations from intended interventions; **D4 – High risk:** outcome assessors not blinded and outcomes included subjective measures; **D5 – High risk:** selective outcome reporting likely due to absence of prospective protocol.

**Supplementary Table 3:** Domain-specific RoB-2 judgments**.**


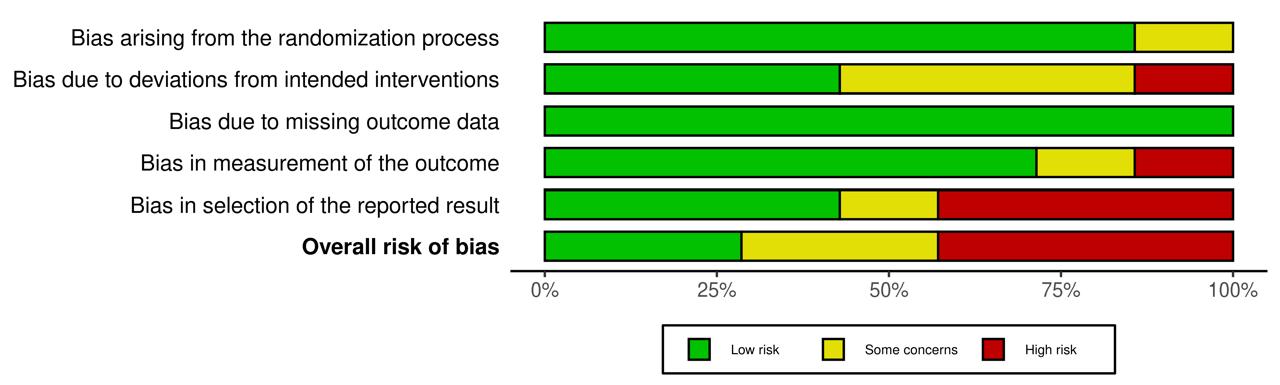

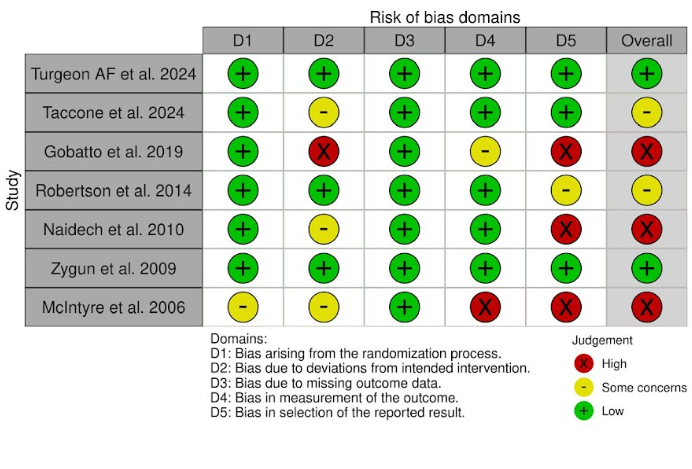


B

A

**Supplementary Figure 1**: The risk of bias (A) and traffic light plot (B) for the included trials.


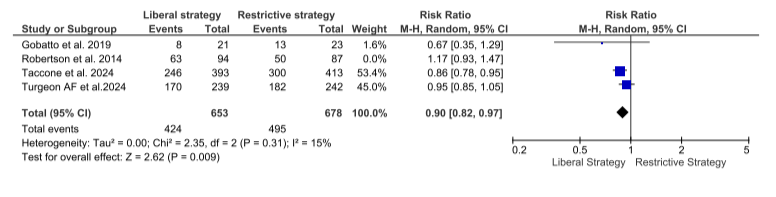


**Supplementary Figure 2:** The impact of different transfusion strategies on unfavorable Glasgow Outcome Scale (GOS) scores after sensitivity analysis.


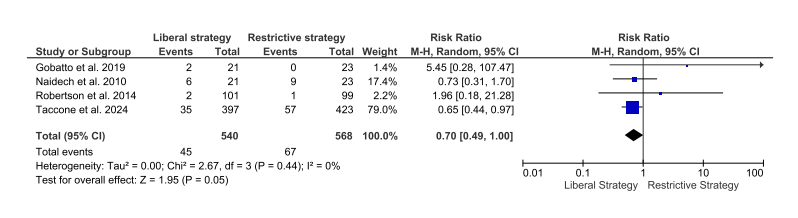


**Supplementary Figure 3:** The impact of different transfusion strategies on stroke.


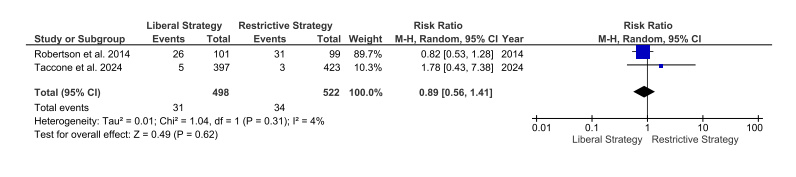


**Supplementary Figure 4:** The impact of different transfusion strategies on brain hypoxia.


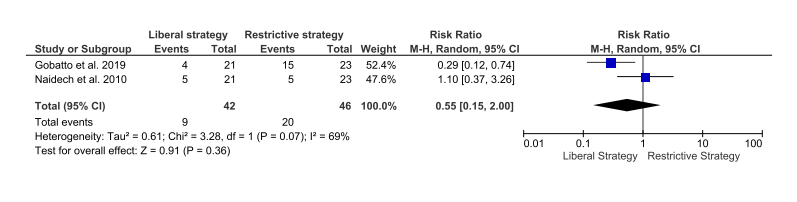


**Supplementary Figure 5:** The impact of different transfusion strategies on vasospasm.


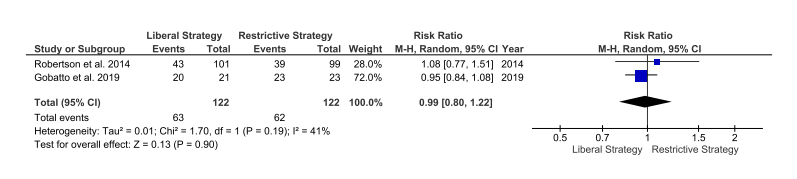


**Supplementary Figure 6:** The impact of different transfusion strategies on intracranial hypertension requiring therapy.

**
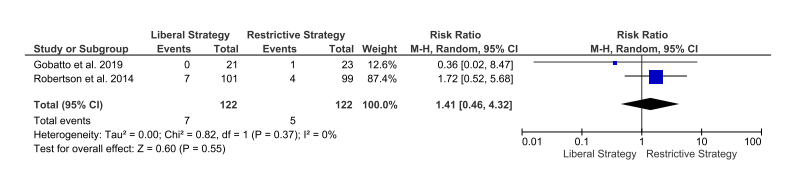
**

**Supplementary Figure 7:** The impact of different transfusion strategies on seizures.

**
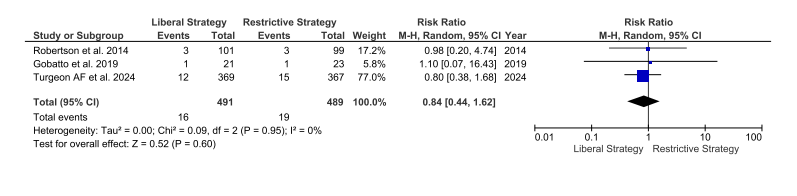
**

**Supplementary Figure 8:** The impact of different transfusion strategies on ventriculitis, meningitis, or brain abscess.


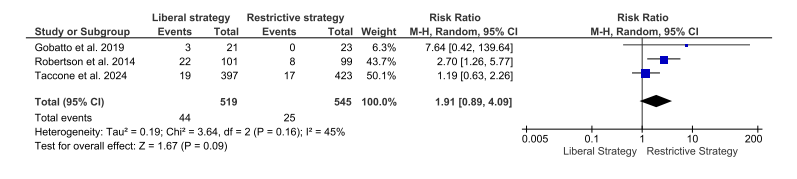


**Supplementary Figure 9:** The impact of different transfusion strategies on thromboembolic events.


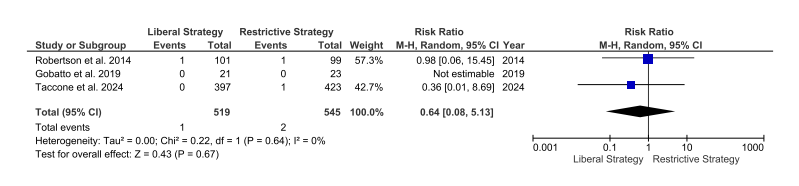


**Supplementary Figure 10:** The impact of different transfusion strategies on acute myocardial infarction (MI).

**
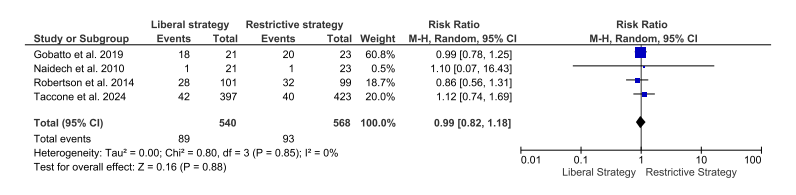
**

**Supplementary Figure 11:** The impact of different transfusion strategies on hypotension.

**
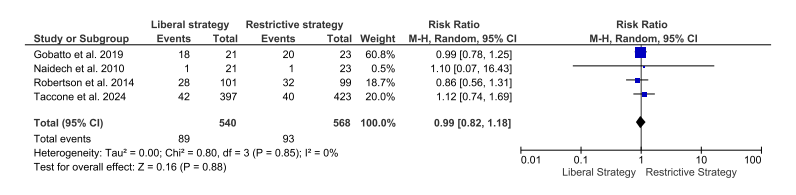
**

**Supplementary Figure 12:** The impact of different transfusion strategies on acute respiratory distress syndrome (ARDS).


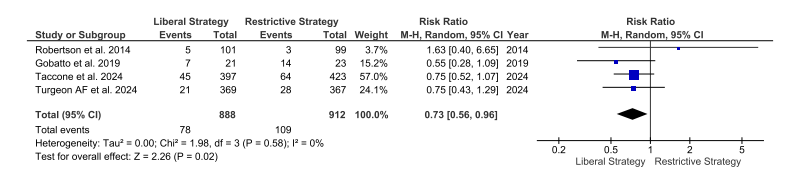


**Supplementary Figure 13:** The impact of different transfusion strategies on sepsis.

**
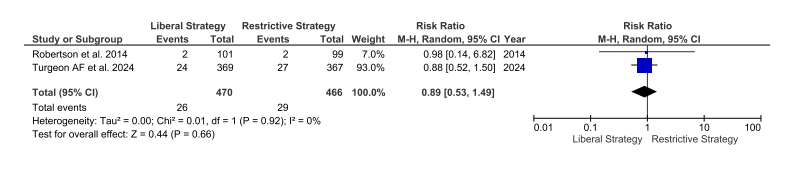
**

**Supplementary Figure 14:** The impact of different transfusion strategies on bacteremia.

**
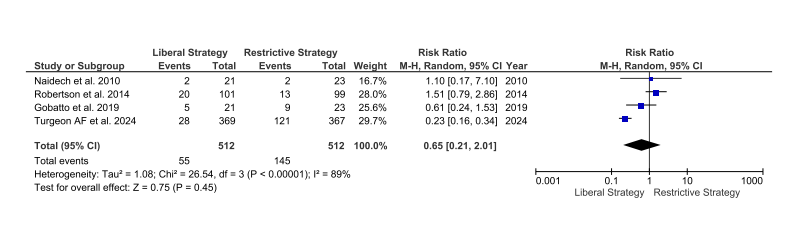
**

**Supplementary Figure 15:** The impact of different transfusion strategies on pneumonia.


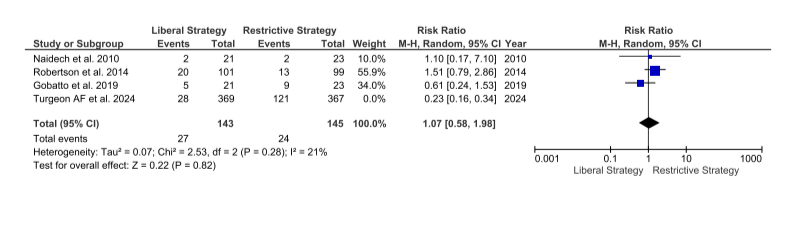


**Supplementary Figure 16:** The impact of different transfusion strategies on pneumonia after sensitivity analysis.


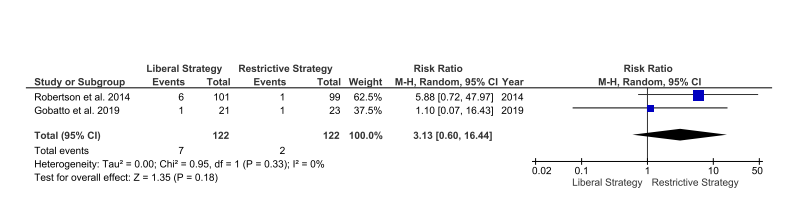


**Supplementary Figure 17:** The impact of different transfusion strategies on pulmonary embolism (PE).


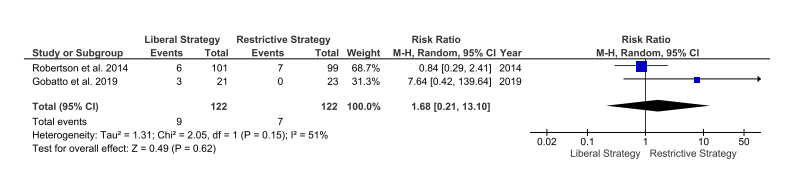


**Supplementary Figure 18:** The impact of different transfusion strategies on urinary tract infections (UTIs).
